# Knowledge and use of antibiotics in six ethnic groups: the HELIUS study

**DOI:** 10.1186/s13756-019-0636-x

**Published:** 2019-12-06

**Authors:** Emelie C. Schuts, Eline van Dulm, Anders Boyd, Marieke B. Snijder, Suzanne E. Geerlings, Maria Prins, Jan M. Prins

**Affiliations:** 1 Department of Internal Medicine, Division of Infectious Diseases, Amsterdam UMC, University of Amsterdam, Meibergdreef 9, 1105 AZ Amsterdam, The Netherlands; 2 Department of Infectious Diseases, Public Health Service Amsterdam, Nieuwe Achtergracht 100, 1018 WT Amsterdam, The Netherlands; 3 INSERM, Sorbonne Université, Institut Pierre Louis d’Épidémiologie et de Santé Publique, Paris, France; 4 Department of Public Health, Amsterdam UMC, University of Amsterdam, Amsterdam Public Health Research Institute, Amsterdam, The Netherlands; 5 Department of Clinical Epidemiology, Biostatistics and Bioinformatics, Amsterdam UMC, University of Amsterdam, Amsterdam Public Health Research Institute, Amsterdam, The Netherlands

**Keywords:** Antibiotics, Antibiotic knowledge, Antibiotic use, Ethnic groups

## Abstract

**Background:**

The increase of antimicrobial resistance, mainly due to increased antibiotic use, is worrying. Preliminary evidence suggests that antibiotic use differs across ethnic groups in the Netherlands, with higher use in people of non-Dutch origin. We aimed to determine whether appropriate knowledge and use of antibiotics differ by ethnicity and whether knowledge on antibiotics is associated with antibiotic use.

**Methods:**

We performed a cross-sectional study analyzing baseline data (2011–2015) from a population-based cohort (HELIUS study), which were linked to data from a health insurance register. We included 21,617 HELIUS participants of South-Asian Surinamese, African-Surinamese, Turkish, Moroccan, Ghanaian, and Dutch origin. Fifteen thousand seven participants had available prescription data from the Achmea Health Data-base (AHD) in the year prior to their HELIUS study visit. Participants were asked five questions on antibiotic treatment during influenza-like illness, pneumonia, fever, sore throat and bronchitis, from which higher versus lower antibiotic knowledge level was determined. Number of antibiotic prescriptions in the year prior to the HELIUS study visit was used to determine antibiotic use.

**Results:**

The percentage of individuals with a higher level of antibiotic knowledge was lower among all ethnic minority groups (range 57 to 70%) compared to Dutch (80%). After correcting for baseline characteristics, including medical conditions, first-generation African Surinamese and Turkish migrants received a significantly lower number of antibiotic prescriptions compared to individuals of Dutch origin. Only second-generation Ghanaian participants received more prescriptions compared to Dutch participants (aIRR 2.09, 95%CI 1.06 to 4.12). Higher level of antibiotic knowledge was not significantly associated with the number of prescriptions (IRR 0.92, 95%CI 0.85 to 1.00).

**Conclusions:**

Levels of antibiotic knowledge varied between ethnic groups, but a lower level of antibiotic knowledge did not correspond with a higher number of antibiotic prescriptions.

## Background

The emergence of antimicrobial resistance, along with the steady decline in antibiotic development, has been identified as a major health threat for the coming decade by the World Health Organization (WHO). Increase in antibiotic use is the main reason for this development [1] and as such, antibiotics should only be prescribed when there is a clear indication for use.

A recent meta-analysis showed a higher prevalence of antimicrobial resistance among migrants in Europe [2]. There is preliminary evidence in the Netherlands that the use of antibiotics also differs across ethnic groups, with a higher use of antibiotics among people of non-Dutch origin [3]. The reason for this difference, however, is unclear. It could be explained by increased incidence of bacterial infections, but, to the best of our knowledge, there is no evidence to support this hypothesis. Alternatively, knowledge about antibiotic use might vary across ethnic groups. As expectations and knowledge of the patient could potentially drive a physician’s decision to prescribe antibiotics, receiving prescriptions could also differ between ethnic groups [4–6]. There are also cultural-specific approaches to dealing with authority, being the physician in this setting, which have explained differences in antibiotic use between countries [7].

The HELIUS (Healthy life in an Urban Setting) study is a large-scale, population-based cohort study among different ethnic groups, which was established with the aim to investigate mechanisms underlying the impact of ethnicity on communicable and non-communicable diseases [8, 9]. In 2018, approximately 13% of the population of the Netherlands was of non-Western origin [10]. The largest non-Western population groups were individuals of Turkish (2.4%), Moroccan (2.3%) and Surinamese (2.0%) descent [10]. In Amsterdam, approximately 36% of the population in 2018 was of non-Western descent [11]. The ethnic groups included in the HELIUS study are the largest ethnic minority groups of Amsterdam [9]. Amongst other data, data on antibiotic knowledge were collected. We were able to link these data at the individual level to data from a health insurance register on recent antibiotic use.

This study then provides a unique opportunity to determine whether knowledge about and use of antibiotics vary between ethnic groups, and if so, whether differences in antibiotic use can be attributed to differences in knowledge about antibiotics. We hypothesized that antibiotic use differs among ethnic groups as a result of differences in knowledge.

## Methods

### Study population and design

The HEalthy LIfe in an Urban Setting (HELIUS) study is a multiethnic cohort study conducted in Amsterdam, which focuses on cardiovascular disease (e.g. diabetes), mental health (e.g. depressive disorders), and infectious diseases [8, 9]. In brief, baseline data collection took place in 2011–2015 and included people aged 18 to 70 years of Dutch, Surinamese, Ghanaian, Moroccan, and Turkish origin. A random sample of participants, stratified by ethnic origin, was taken from the municipality register of Amsterdam. Participants filled in an extensive self-administered questionnaire (variables included in the questionnaire are described elsewhere) [9] and underwent a physical examination during which biological samples were obtained [9]. No information was provided regarding appropriate antibiotic use. Between 2011 and 2015, 24,789 persons were included. Data collection procedures have been previously described in detail [9]. Both questionnaire data and physical examination data were available for 22,165 participants. The HELIUS study was conducted in accordance with the Declaration of Helsinki and was approved by the AMC Ethical Review Board. All participants provided written informed consent.

Ethnicity was defined according to the country of birth of the participant as well as that of their parents [12]. Specifically, a participant is considered to be of non-Dutch ethnic origin if they fulfill either of the following criteria (i): they were born abroad and had at least one parent born abroad (first generation) or (ii) they were born in Netherlands but both their parents were born abroad (second generation). Dutch participants were born in the Netherlands and had both parents who were born in the Netherlands. After HELIUS data collection, the Surinamese group were further classified according to self-reported ethnic origin (obtained by questionnaire), into ‘African Surinamese’, ‘South-Asian Surinamese’, ‘Javanese Surinamese’ and ‘other/unknown Surinamese’.

### Data linkage

Permission to link participants’ individual data to outside health registries was asked in the written informed consent form [8]. Of the 22,165 HELIUS participants, 19,895 agreed. HELIUS data of these individuals were linked to reimbursement data from the Achmea insurance company (Achmea Health Database, AHD) from 2010 until 2015. The AHD, obtained from the largest health insurance company in Amsterdam, contains all healthcare expenditures of every insured participant, including medications. A trusted third party linked data on reimbursed antibiotic prescriptions using an encrypted social security number and returned data without any identifying information. Procedures were in accordance with the General Data Protection Regulation [13].

### Inclusion and exclusion criteria for present study

Of the 22,165 participants, we excluded those of Javanese Surinamese or other/unknown Surinamese origin and those with another/unknown ethnic origin because of small participant numbers. For analyses on antibiotic use, we included those who gave permission for data linkage and could be linked to the AHD. To reduce bias for individuals with short-term insurance, we excluded those who were insured with Achmea for less than 365 days in the year preceding their HELIUS study visit.

### Outcome variables

The primary outcomes were level of antibiotic knowledge and antibiotic use during the year prior to the HELIUS visit. Level of antibiotic knowledge was based on five questions, used in other studies [4, 6, 14], which asked the perceived necessity (yes/no) for antibiotic treatment during influenza-like illness, pneumonia, fever, sore throat and bronchitis. Using these questions, we created an overall knowledge score of antibiotic use by summing the total number of correct responses, resulting in a score ranging from 0 to 5. A two-parameter logistic regression model was fitted to the five binary items based on the assumptions of item response theory (see Additional file 1). From this model, “higher” and “lower” levels of antibiotic knowledge were defined by a knowledge score of ≥4 and < 4, respectively.

Antibiotic use was obtained from linked AHD data and was based on the total number of reimbursed antibiotics (classified by ATC code J01; anti-infectives for systemic use) dispensed by community pharmacies from 2010 until 2015. We evaluated antibiotic use (yes/no) in the year prior to the HELIUS study visit, as well as the number of antibiotic prescriptions over the past year and during the entire insured period.

### Other variables

Independent variables were obtained from the HELIUS study questionnaire (migration generation; sex; age; level of education; marital status; self-reported medical conditions; smoking; alcohol consumption; difficulty with the Dutch language and perceived health) and physical examination (body mass index (BMI, kg/m^2^)). Variables on antibiotic-related behavior were: not having finished antibiotic treatment; having saved antibiotics for later; and ever having asked the general practitioner (GP) for antibiotics. Definitions and grouping of variables are extensively described elsewhere [8].

### Statistical analyses

Sociodemographics, health status, antibiotic knowledge level and questions on antibiotic use were presented by ethnicity. To assess selection bias resulting from AHD data linkage, the same variables were compared between participants who were successfully versus unsuccessfully linked. Comparisons between ethnic groups were made using a Pearson’s χ^2^ or Fisher exact test for categorical data and Kruskal-Wallis rank test for continuous variables.

Analysis on level of antibiotic knowledge included all HELIUS participants with available data. Odds ratios (OR) comparing levels of antibiotic knowledge across determinants and their 95% confidence intervals (CI) were estimated using logistic regression. All variables with an associated *p*-value < 0.2 in univariable analyses were included in a full multivariable model and variables with a *p*-value above this level were removed in backwards-stepwise fashion. Given that the research aim was to determine differences between ethnicity, ethnic groups were forced in all models. This multivariable approach was chosen to not only assess other variables associated with antibiotic knowledge, but also to understand the extent of confounding bias when assessing the relationship between ethnicity and outcome variables.

Analysis on antibiotic use in the year prior to HELIUS study visit included all HELIUS participants who were linked to the AHD and were insured for at least 365 days with Achmea in the year prior to their HELIUS study visit. Determinants for having received ≥1 antibiotic prescription were assessed using logistic regression. The same multivariable approach as above was used for this outcome. We also compared antibiotic use during the entire period insured at Achmea versus the year prior to HELIUS study visit to assess differences when considering longer time periods.

Determinants for the total number of antibiotic prescriptions were then evaluated. As this outcome contained a high proportion of zero values and was over-dispersed, we used a zero-inflated negative binomial regression model. This model contains two parts: one accounting for zero values in the count distribution (zero-inflated) and another accounting for the over-dispersed count distribution (negative binomial). Covariates for the zero-inflated part were determined a priori from the risk-factor analysis on ≥1 antibiotic prescription. Covariates for the negative binomial part were selected from covariates with a *p*-value < 0.2 in univariable analyses and variables above this *p*-value were removed in backwards-stepwise fashion. Incidence risk ratios (IRR) comparing the number of antibiotics prescribed over the past year across levels of determinants were estimated from this model.

Multicollinearity was verified using variance inflation factors, while any variable with an inflation factor of ≥4 was considered multicollinear and excluded from the model. To understand whether the association between ethnicity and outcome was modified by demographic variables, interaction between ethnicity and other demographic variables was also assessed in all multivariable models.

The three variables involving antibiotic-related behavior were not initially considered in the final multivariable models. To assess whether ethnic differences in antibiotic use could be explained by patterns of antibiotic-related behavior, additional multivariable models including these variables were constructed for the endpoints (i) having received ≥1 antibiotic prescription and (ii) total number of antibiotic prescriptions.

**Fig. 1:**
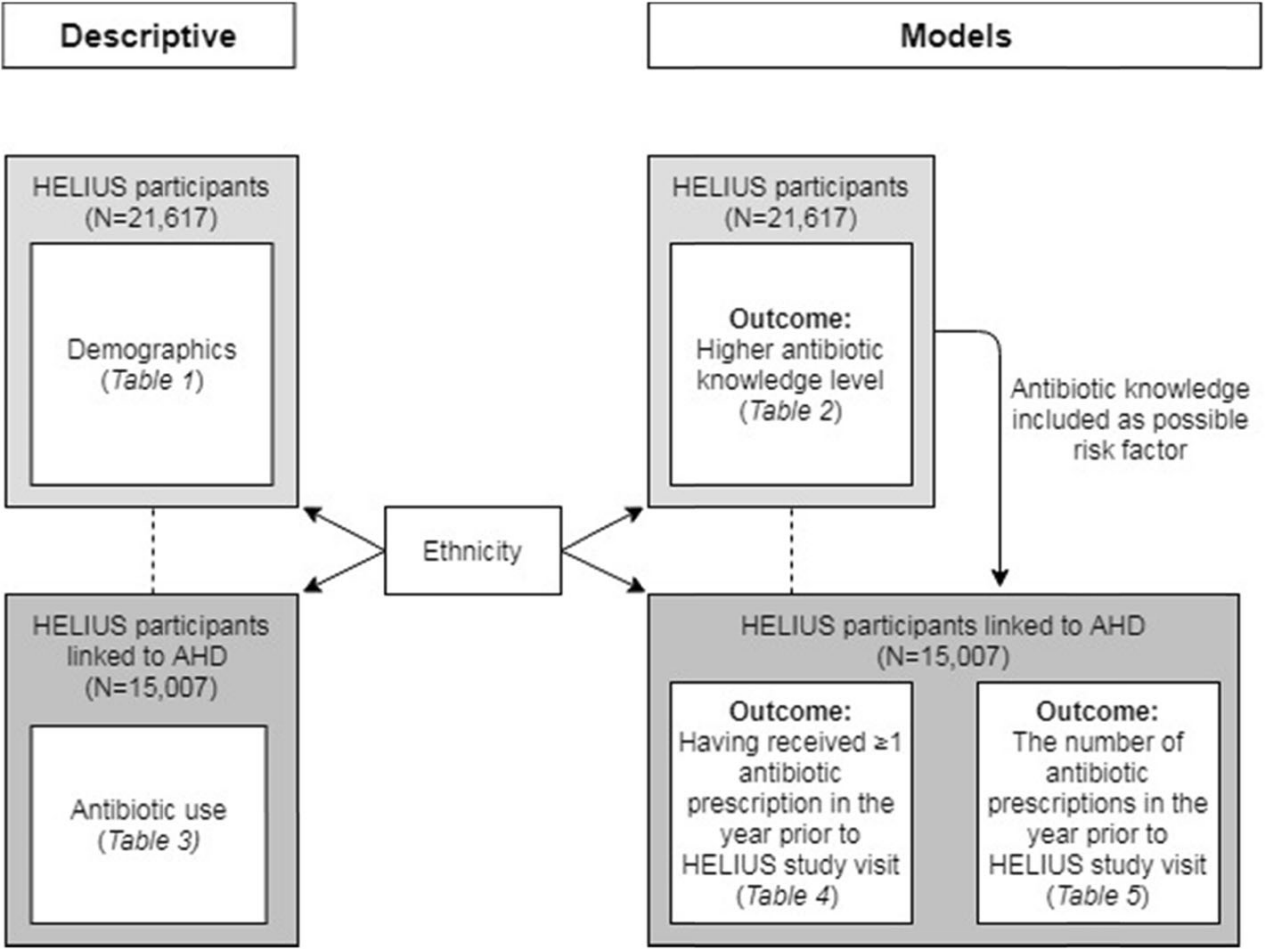
Overview of descriptive analysis and models used in the study. Abbreviations: HELIUS – Healthy Life in an Urban Setting; AHD – Achmea Health Database

Figure 1 provides an overview of all descriptive analysis and modeling used in the study. Significance was determined using a *p*-value < 0.05. All analyses were conducted with Stata 13.1 (StataCorp., College Station, Texas, USA).

## Results

### Participants

Of the 22,165 HELIUS participants with available data, 21,617 were eligible after applying exclusion criteria. Their baseline characteristics, stratified by ethnicity, are shown in Table 1. Median age of participants was 46 years (IQR 34 to 55) and 58% were women. The proportion of several medical conditions predisposing individuals to antibiotic treatment differed by ethnicity. Of these conditions, South-Asian Surinamese participants had the highest prevalence of self-reported diabetes mellitus (17%) and cerebrovascular accident (CVA) (6%) over the last 12 months. Turkish individuals had more prevalent artery stenosis (10%), severe or chronic fatigue (45%) and respiratory diseases (15%), whereas Ghanaians more frequently reported high blood pressure (33%). Excellent perceived health was reported in 12% of Dutch participants in contrast to 3.3% of Turkish participants.

**Table 1:** Characteristics of the HELIUS study population (*N* = 21,617) by ethnicity

Variables^a^	Ethnicity											
	Dutch (*N* = 4564)		South-Asian Surinamese (*N* = 3043)		African Surinamese (*N* = 4151)		Ghanaian (*N* = 2339)		Turkish (*N* = 3614)		Moroccan (*N* = 3906)	
Sociodemographics												
Female sex	2475	54%	1672	55%	2535	61%	1434	61%	1980	55%	2392	61%
Age in years, median (IQR)	47	(34–58)	48	(35–56)	50	(40–57)	47	(38–53)	42	(31–50)	40	(30–50)
Migration generation												
1st generation	N.A.	N.A.	2328	77%	3468	84%	2231	95%	2544	70%	2680	69%
2nd generation	N.A.	N.A.	715	23%	683	16%	108	4.6%	1080	30%	1226	31%
Educational level												
Unknown	25	0.6%	16	0.5%	36	0.9%	42	1.8%	38	1.1%	38	1.0%
No school/elementary school	150	3.3%	437	14%	231	6%	660	28%	1135	31%	1205	31%
Lower vocational/lower secondary school	646	14%	1010	33%	1477	36%	917	39%	889	25%	694	18%
Intermediate vocational/ intermediate secondary school	994	22%	885	29%	1464	35%	578	25%	1020	28%	1294	33%
Higher vocational/university	2749	60%	695	23%	943	23%	142	6%	532	15%	675	17%
Marital status												
Married/registered partnership	1724	38%	1043	34%	766	19%	420	18%	2208	61%	2285	59%
Cohabiting	914	20%	311	10%	441	11%	427	19%	132	3.7%	110	2.8%
Unmarried/never married	1474	32%	1001	33%	2231	54%	779	34%	761	21%	1010	26%
Divorced/separated	356	8%	580	19%	617	15%	656	28%	407	11%	414	11%
Widow/widower	87	1.9%	92	3.0%	65	1.6%	23	1.0%	90	2.5%	69	1.8%
Health status												
Self-reported medical conditions (previous 12 months)												
Diabetes mellitus	102	2.2%	521	17%	419	10%	185	8%	336	9%	389	10%
CVA/one-sided loss of bodily function ≤1 day	160	3.5%	212	7%	261	6%	95	4.1%	196	5%	195	5%
MI incl. ≥half hour chest pain or dotter/bypass operation	233	5%	491	16%	440	11%	225	10%	591	16%	476	12%
Severe heart condition	67	1.5%	120	4.0%	105	2.5%	75	3.2%	153	4.3%	58	1.5%
Malignant disorder	103	2.3%	70	2.3%	85	2.1%	33	1.4%	73	2.0%	46	1.2%
Severe or chronic fatigue	633	14%	1032	34%	956	23%	186	8%	1602	45%	1465	38%
High blood pressure	534	12%	720	24%	1230	30%	770	33%	610	17%	546	14%
Artery stenosis	85	1.9%	193	6%	181	4.4%	115	5%	348	10%	200	5%
Respiratory diseases	345	8%	433	14%	354	9%	117	5%	556	15%	446	11%
Serious/persistent intestinal disorders	249	5%	248	8%	308	7%	70	3.0%	433	12%	391	10%
Psoriasis	136	3.0%	168	6%	128	3.1%	71	3.1%	154	4.3%	121	3.1%
(Chronic) eczema	420	9%	406	13%	370	9%	71	3.1%	471	13%	423	11%
Incontinence	309	7%	326	11%	342	8%	108	4.7%	464	13%	300	8%
Body Mass Index (kg/m^2^), median (IQR)	24.1	(21.9–26.7)	25.7	(23.2–28.8)	27.0	(23.9–30.8)	27.9	(25.0–31.2)	27.9	(24.6–31.7)	27.0	(23.9–30.7)
Smoking												
Yes	1129	25%	861	28%	1309	32%	104	4.5%	1240	35%	525	13%
No, never	1689	37%	1758	58%	2016	49%	2027	87%	1700	47%	2874	74%
No, but ever	1737	38%	413	14%	805	19%	191	8%	648	18%	492	13%
Alcohol consumption												
Never	297	7%	1072	35%	1002	24%	806	35%	2414	67%	3265	84%
Not in previous 12 months	110	2.4%	251	8%	292	7%	408	18%	358	10%	338	9%
Monthly or less	436	10%	758	25%	1242	30%	508	22%	367	10%	127	3.3%
2–4 times per month	894	20%	541	18%	873	21%	291	13%	257	7%	87	2.2%
2–3 times per week	1413	31%	262	9%	439	11%	193	8%	132	3.7%	56	1.4%
≥ 4 times per week	1408	31%	147	5%	272	7%	109	4.7%	57	1.6%	16	0.4%
Difficulty with Dutch language												
Yes	N.A.	N.A.	711	23%	520	13%	1926	83%	2136	60%	1774	46%
Perceived health												
Excellent	541	12%	162	5%	303	7%	226	10%	117	3.3%	166	4.3%
Very good	1381	30%	310	10%	571	14%	458	20%	383	11%	384	10%
Good	2205	48%	1623	53%	2335	56%	1180	51%	1871	52%	1871	48%
Mediocre	402	9%	811	27%	834	20%	383	16%	921	26%	1223	31%
Bad	28	0.6%	131	4.3%	101	2.4%	88	3.8%	307	9%	241	6%
Antibiotics												
Knowledge concerning antibiotics^b^												
Antibiotics effective for influenza	324	7%	554	19%	592	15%	658	29%	744	21%	648	18%
Antibiotics effective for pneumonia	4166	92%	2304	77%	3114	77%	1312	58%	2587	73%	2741	73%
Antibiotics effective for fever^c^	689	15%	552	19%	679	17%	586	26%	898	26%	614	17%
Antibiotics effective for sore throat^c^	672	15%	760	26%	1089	27%	720	32%	1203	34%	978	26%
Antibiotics effective for bronchitis	2246	50%	1385	50%	1862	46%	919	41%	1235	35%	1485	40%
Higher level of antibiotic knowledge^d^	3638	80%	1996	68%	2737	69%	1248	57%	2128	62%	2528	70%
Did not finish antibiotic treatment												
Yes, regularly	5	0.1%	49	1.6%	44	1.1%	48	2.1%	41	1.2%	44	1.1%
Yes, occasionally	332	7%	312	10%	527	13%	174	8%	424	12%	445	12%
Always finished or no antibiotics	4203	93%	2646	88%	3524	86%	2053	90%	3104	87%	3361	87%
Saved antibiotics for later												
Yes, regularly	2	0.0%	7	0.2%	9	0.2%	5	0.2%	10	0.3%	6	0.2%
Yes, occasionally	37	0.8%	45	1.5%	68	1.7%	62	2.7%	60	1.7%	46	1.2%
No, never	297	7%	304	10%	492	12%	146	6%	387	11%	430	11%
Not applicable (no antibiotics)	4203	93%	2646	88%	3524	86%	2053	91%	3104	87%	3361	87%
Ever asked GP for antibiotics												
Yes, regularly	38	0.8%	34	1.1%	26	0.6%	36	1.6%	67	1.9%	71	1.9%
Yes, occasionally	824	18%	491	16%	607	15%	401	18%	734	21%	634	17%
No, never	3674	81%	2482	83%	3441	84%	1835	81%	2744	77%	3074	81%

Missing data, *n*: marital status 128; diabetes 78; stroke 55; myocardial infarction 33; heart condition 83; malignant disorders 137; fatigue 145; high blood pressure 101; artery stenosis 140; respiratory diseases 115; bowel diseases 115; psoriasis 98; eczema 117; incontinence 126; BMI 23; smoking 107; alcohol 127; perceived health 61; AB effective for influenza 614; AB effective for pneumonia 477; AB effective for fever 685; AB effective for sore throat 625; AB effective for bronchitis 663; asked GP for AB 414; did not finish treatment 292; saved AB 325

*Abbreviations*: *IQR* Inter Quartile Range, *CVA* Cerebro Vascular Accident, *MI* Myocardial infarction, *N.A.* Not Applicable, *GP* General Practitioner

*N.A.* Not applicable (categories not applicable due to Dutch ethnicity)

^a^All variables are reported as n (%), unless otherwise indicated

^b^Answered “yes” to the statements below

^c^The Dutch General Practitioners guidelines (and those of other European countries) advise against the use of antibiotics for fever in general or sore throat, as they usually constitute viral infections, with only a few exceptions in both cases. Therefore, antibiotics are in general not appropriate for these conditions

^d^Based on a summed score with cutoff determined by an Item Response Theory model (≥4 out of 5 antibiotic knowledge questions correctly answered was considered as having a higher level of knowledge)

### Ethnic differences in antibiotic knowledge

In several ethnic groups, there were substantial proportions of individuals reporting the need to be treated with antibiotics for illnesses without indication, as shown in Table 1. The number of people reporting to have been treated with antibiotics and not having regularly completed their antibiotic treatment was low across all ethnic groups, ranging from 0.1% in Dutch participants to 2.1% in Ghanaian participants. Few individuals regularly saved their antibiotics for later use, ranging from < 0.1% in Dutch participants to 0.3% in Turkish participants. The percentage of participants having regularly asked their GP for antibiotics ranged from 0.6% in African Surinamese participants to 1.9% in Turkish and Moroccan participants.

As shown in Table 2, there was a significantly lower odds of individuals with higher level of antibiotic knowledge among all non-Dutch ethnic groups compared to Dutch individuals (overall *p* < 0.001) (Table 2). Across all non-Dutch groups, second-generation participants had a higher level of antibiotic knowledge than first-generation participants; however, results remained significantly lower compared to the Dutch group.

**Table 2:** Variables associated with higher antibiotic knowledge in HELIUS study population (*N* = 21,617) (logistic regression analysis)

	Univariable			Multivariable (*N* = 20,081^a^)#		
	OR	(95% CI)	*P*-values	aOR	(95% CI)	*P*-values
Sociodemographics						
Ethnicity			<.001			<.001
Dutch	Ref			Ref		
South-Asian Surinamese						
1st generation	0.49	0.44–0.55		0.53	0.47–0.60	
2nd generation	0.56	0.47–0.67		0.60	0.50–0.73	
African Surinamese						
1st generation	0.51	0.46–0.57		0.53	0.47–0.59	
2nd generation	0.75	0.62–0.91		0.79	0.64–0.96	
Ghanaian						
1st generation	0.31	0.27–0.34		0.31	0.27–0.35	
2nd generation	0.64	0.41–0.98		0.74	0.47–1.18	
Turkish						
1st generation	0.35	0.31–0.39		0.40	0.36–0.45	
2nd generation	0.56	0.48–0.65		0.62	0.53–0.74	
Moroccan						
1st generation	0.51	0.45–0.57		0.56	0.50–0.63	
2nd generation	0.71	0.61–0.83		0.75	0.63–0.89	
Female sex	1.18	1.11–1.25	<.001	1.32	1.23–1.40	<.001
Age			<.001			<.001
< 25 years	Ref			Ref		
25–34 years	1.24	1.01–1.34		1.32	1.16–1.50	
35–44 years	0.99	0.81–1.05		1.30	1.14–1.49	
45–54 years	0.85	0.71–0.91		1.19	1.04–1.37	
55–64 years	0.95	0.78–1.02		1.26	1.08–1.45	
≥ 65 years	1.06	0.84–1.22		1.15	0.95–1.39	
Educational level			<.001			
Unknown	Ref					
No school/elementary school	1.10	0.79–1.55				
Lower vocational/lower secondary school	1.27	0.91–1.77				
Intermediate vocational/ intermediate secondary school	1.54	1.11–2.15				
Higher vocational/university	2.06	1.47–2.87				
Marital status			<.001			
Married/registered partnership	Ref					
Cohabiting	1.15	1.04–1.27				
Unmarried/never married	1.08	1.01–1.16				
Divorced/separated	0.81	0.74–0.89				
Widow/widower	1.10	0.88–1.36				
Health status						
Self-reported medical conditions (previous 12 months)						
Diabetes mellitus	0.70	0.63–0.77	<.001			
CVA/one-sided loss of bodily function ≤1 day	0.85	0.75–0.97	.015			
MI incl. ≥half hour chest pain or dotter/bypass operation	0.71	0.65–0.77	<.001	0.89	0.81–0.98	.017
Severe heart condition	0.61	0.52–0.73	<.001			
Malignant disorder	0.97	0.78–1.20	.752			
Severe or chronic fatigue	0.80	0.75–0.85	<.001	0.89	0.82–0.95	.001
High blood pressure	0.78	0.73–0.84	<.001			
Artery stenosis	0.69	0.61–0.78	<.001			
Respiratory diseases	0.68	0.62–0.75	<.001	0.80	0.72–0.88	<.001
Serious/persistent intestinal disorders	0.88	0.79–0.98	.024			
Psoriasis	0.76	0.66–0.89	.001			
(Chronic) eczema	0.93	0.84–1.02	.121			
Incontinence	0.84	0.76–0.93	.001			
Body Mass Index			<.001			.001
< 18.5	Ref			Ref		
18.5–25	1.03	0.81–1.31		1.02	0.80–1.31	
25–30	0.80	0.63–1.01		0.96	0.75–1.24	
30–40	0.67	0.52–0.85		0.86	0.66–1.11	
≥ 40	0.58	0.43–0.78		0.78	0.57–1.08	
Smoking			<.001			
Yes	Ref					
No, never	1.03	0.96–1.11				
No, but ever	1.19	1.09–1.30				
Alcohol usage			<.001			
Never	Ref					
Not in previous 12 months	0.89	0.80–1.00				
Monthly or less	1.10	1.01–1.20				
2–4 times per month	1.27	1.16–1.40				
2–3 times per week	1.35	1.22–1.49				
≥ 4 times per week	1.59	1.42–1.78				
Difficulty with Dutch language			<.001			
No	Ref					
Yes	0.65	0.61–0.69				
Not applicable	1.70	1.56–1.85				
Perceived health			<.001			
Excellent	Ref					
Very good	0.97	0.85–1.12				
Good	0.83	0.73–0.93				
Mediocre	0.63	0.56–0.72				
Bad	0.48	0.40–0.57				
Antibiotics						
Ever asked GP for antibiotics			<.001			<.001
No, never	Ref			Ref		
Yes, regularly	0.51	0.40–0.65		0.60	0.46–0.77	
Yes, occasionally	0.57	0.53–0.61		0.59	0.55–0.64	
Did not finish treatment			<.001			<.001
Always finished or no antibiotics	Ref			Ref		
Yes, regularly	0.51	0.39–0.67		0.71	0.54–0.94	
Yes, occasionally	0.73	0.66–0.80		0.80	0.73–0.88	

*Abbreviations*: *OR* Odds Ratio, *aOR* adjusted Odds Ratio, *CI* Confidence Interval, *CVA* Cerebro Vascular Accident, *MI* Myocardial infarction, *GP* General Practitioner

^a^Fewer observations in the multivariable model than in the total study population were due to missing observations on certain covariates

#We found significant interactions between ethnicity and sex (*p* = 0.007) and ethnicity and age (*p* = 0.047)

In multivariable analysis, all ethnic minority groups had lower odds for higher level of antibiotic knowledge compared to Dutch (overall *p* < 0.001), although the effect for second-generation Ghanaian participants was not statistically significant. The odds for higher level of antibiotic knowledge were higher in all age groups > 25 years of age (except for those ≥65) when compared to ≤25 years of age. Furthermore, women had a significantly higher odds of having a higher level of antibiotic knowledge compared to males. Lower odds for a higher level of antibiotic knowledge were found for the following medical conditions: myocardial Infarction (MI), severe or chronic fatigue, respiratory diseases and having a BMI ≥25. Lower odds for higher level of antibiotic knowledge were also seen among individuals who regularly or occasionally requested antibiotics from their GP or who regularly or occasionally did not finish treatment.

### Ethnic difference in antibiotic use

Of the 19,895 HELIUS participants consenting to link their data to other health registries, 15,461 were linked to the AHD (77.7%). Of these 15,461 participants, 15,007 (97%) were insured for ≥365 days in the year prior to their HELIUS study visit. Additional file 2: Table S1 shows the characteristics of the study participants linked versus not linked to the AHD. Participants present in the AHD register had a lower level of education, higher prevalence of medical conditions, and less often had higher levels of antibiotic knowledge.

Table 3 describes antibiotic use according to ethnicity for participants registered in the AHD. In total, 31,530 antibiotic prescriptions were recorded over the study period. The proportion of participants receiving ≥1 antibiotic prescription in the year prior to their HELIUS study visit was highest among first-generation Turkish participants (25%) and was comparably high among second-generation Ghanaian and first-generation Moroccan participants (both 25%). The proportion of participants receiving ≥1 antibiotic prescription in the year prior to the HELIUS study visit was lowest in Dutch and second generation South-Asian Surinamese participants (both 16%).

**Table 3:** Antibiotic use in participants linked to AHD (*N* = 15,007) stratified by ethnicity

	Ethnicity										
	Dutch	South-Asian Surinamese		African Surinamese		Ghanaian		Turkish		Moroccan	
	(*N* = 2071)	1st gen (*N* = 1645)	2nd gen (*N* = 452)	1st gen (*N* = 2334)	2nd gen (*N* = 432)	1st gen (*N* = 1789)	2nd gen (*N* = 84)	1st gen (*N* = 2102)	2nd gen (*N* = 776)	1st gen (*N* = 2119)	2nd gen (*N* = 857)
Duration of insurance at Achmea (in years) between 2010 and 2015, median (IQR)	6.0 (4.0–6.0)	6.0 (6.0–6.0)	6.0 (4.0–6.0)	6.0 (6.0–6.0)	6.0 (5.0–6.0)	6.0 (6.0–6.0)	6.0 (4.0–6.0)	6.0 (96.0–6.0)	6.0 (5.0–6.0)	6.0 (6.0–6.0)	6.0 (4.0–6.0)
Within year prior to HELIUS study visit											
Participants with ≥1 ABP	16%	22%	16%	17%	17%	22%	25%	25%	19%	25%	17%
Number of ABP among all participants included in the AHD											
Mean	0.26	0.39	0.26	0.28	0.28	0.33	0.55	0.40	0.34	0.41	0.28
Median (IQR)	0.00 (0.0–0.0)	0.00 (0.0–0.0)	0.00 (0.0–0.0)	0.00 (0.0–0.0)	0.00 (0.0–0.0)	0.00 (0.0–0.0)	0.00 (0.0–0.5)	0.00 (0.0–1.0)	0.00 (0.0–0.0)	0.00 (0.0–1.0)	0.00 (0.0–0.0)
Number of ABP among participants with ≥1 ABP											
Mean	1.66	1.75	1.59	1.58	1.64	1.51	2.19	1.57	1.78	1.64	1.63
Median (IQR)	1.00 (1.0–2.0)	1.00 (1.0–2.0)	1.00 (1.0–2.0)	1.00 (1.0–2.0)	1.00 (1.0–2.0)	1.00 (1.0–2.0)	2.00 (1.0–3.0)	1.00 (1.0–2.0)	1.00 (1.0–2.0)	1.00 (1.0–2.0)	1.00 (1.0–2.0)
During entire insured period											
Participants with ≥1 ABP	51%	63%	53%	56%	51%	62%	49%	69%	59%	67%	54%
Number of ABP per year among all participants included in the AHD											
Mean	0.31	0.46	0.30	0.31	0.30	0.35	0.34	0.44	0.38	0.43	0.33
Median (IQR)	0.17 (0.0–0.3)	0.17 (0. 0–0.6)	0.17 (0. 0–0.3)	0.17 (0.0–0.3)	0.17 (0.0–0.3)	0.17 (0.0–0.5)	0.00 (0.0–0.7)	0.25 (0.0–0.7)	0.17 (0.0–0.5)	0.17 (0.0–0.6)	0.17 (0.0–0.5)

*Abbreviations*: *ABP* Antibiotic Prescription, *IQR* Inter Quartile Range

When considering the entire period during which participants were insured at Achmea prior to the HELIUS study visit (median 6.0 years, IQR 5.0 to 6.0), the proportion of participants receiving ≥1 antibiotic prescription was highest among first generation Turkish participants (69%) and lowest in second-generation Ghanaian participants (49%). The mean number of prescriptions during the entire insured period was comparable to the mean number of prescriptions in the year prior to HELIUS study visit for all ethnic groups (Table 2).

### Determinants of antibiotic use and number of prescriptions

Table 4 shows the results from the analysis on the association between ethnicity and having received ≥1 antibiotic prescription in the year prior to the HELIUS study visit. Differences across ethnic groups were observed overall for any antibiotic prescription in both univariable (*p* < 0.001) and multivariable analysis (*p* < 0.001). In multivariable analysis, compared to Dutch individuals, first and second generation Ghanaian individuals and first-generation Moroccan individuals had significantly higher odds of receiving ≥1 antibiotic prescription. Adding variables on antibiotic-related behavior and level of antibiotic use knowledge to the multivariable model did not change these associations.

**Table 4:** Variables associated with having received ≥1 antibiotic prescription in the year prior to HELIUS visit in participants linked to AHD (*N* = 15,007) (logistic regression analysis)

	Univariable			Multivariable excluding variables on antibiotic-related behavior			Multivariable including variables on antibiotic-related behavior		
	OR	(95% CI)	*P*-values	aOR	(95% CI)	*P*-values	aOR	(95% CI)	*P*-values
Sociodemographics									
Ethnicity			<.001			<.001			.004
Dutch	Ref			Ref			Ref		
South-Asian Surinamese									
1st generation	1.57	1.33–1.85		1.05	0.86–1.27		1.04	0.85–1.26	
2nd generation	1.05	0.79–1.38		0.95	0.71–1.28		0.92	0.68–1.24	
African Surinamese									
1st generation	1.15	0.98–1.35		0.89	0.75–1.07		0.88	0.73–1.05	
2nd generation	1.14	0.87–1.50		1.02	0.76–1.36		0.96	0.71–1.29	
Ghanaian									
1st generation	1.53	1.30–1.81		1.38	1.14–1.68		1.28	1.05–1.56	
2nd generation	1.81	1.09–3.01		1.92	1.12–3.27		1.64	0.94–2.87	
Turkish									
1st generation	1.84	1.58–2.15		1.07	0.88–1.31		1.00	0.82–1.22	
2nd generation	1.27	1.02–1.57		1.02	0.80–1.30		1.01	0.79–1.29	
Moroccan									
1st generation	1.81	1.55–2.11		1.22	1.00–1.49		1.15	0.94–1.41	
2nd generation	1.14	0.92–1.41		0.93	0.73–1.19		0.89	0.69–1.14	
Female sex	1.91	1.75–2.08	<.001	1.77	1.60–1.95	<.001	1.70	1.54–1.88	<.001
Age			<.001						
< 25 years	Ref								
25–34 years	1.04	0.87–1.24							
35–44 years	1.28	1.09–1.50							
45–54 years	1.34	1.15–1.56							
55–64 years	1.42	1.21–1.66							
≥ 65 years	1.59	1.29–1.96							
Educational level			<.001			.005			.001
Unknown	Ref			Ref			Ref		
No school/elementary school	1.41	0.95–2.09		1.55	0.93–2.58		1.55	0.87–2.75	
Lower vocational/lower secondary school	1.05	0.71–1.55		1.50	0.90–2.50		1.47	0.83–2.60	
Intermediate vocational/ intermediate secondary school	0.93	0.63–1.39		1.43	0.86–2.39		1.38	0.78–2.44	
Higher vocational/university	0.66	0.44–0.99		1.18	0.71–1.99		1.12	0.63–2..00	
Marital status			<.001						
Married/registered partnership	Ref								
Cohabiting	0.66	0.56–0.77							
Unmarried/never married	0.78	0.71–0.86							
Divorced/separated	1.14	1.02–1.27							
Widow/widower	1.26	0.98–1.64							
Health status									
Self-reported medical conditions (previous 12 months)									
Diabetes mellitus	1.75	1.56–1.96	<.001	1.29	1.13–1.47	<.001	1.30	1.13–1.48	<.001
CVA/one-sided loss of bodily function ≤1 day	1.38	1.18–1.62	<.001						
MI incl. ≥half hour chest pain or dotter/bypass operation	1.78	1.60–1.98	<.001	1.28	1.13–1.45	<.001	1.24	1.09–1.40	.001
Severe heart condition	1.45	1.18–1.79	.001						
Malignant disorder	1.93	1.52–2.47	<.001	1.33	1.02–1.74	.037			
Severe or chronic fatigue	1.86	1.71–2.02	<.001	1.20	1.08–1.33	.001	1.16	1.04–1.29	.008
High blood pressure	1.36	1.25–1.49	<.001						
Artery stenosis	1.46	1.25–1.70	<.001	0.80	0.67–0.96	.014	0.77	0.64–0.92	.004
Respiratory diseases	2.19	1.96–2.44	<.001	1.66	1.47–1.87	<.001	1.59	1.41–1.81	<.001
Serious/persistent intestinal disorders	1.87	1.64–2.12	<.001	1.24	1.07–1.43	.004	1.22	1.05–1.41	.009
Psoriasis	1.28	1.05–1.56	.015						
(Chronic) eczema	1.30	1.15–1.47	<.001						
Incontinence	2.08	1.85–2.35	<.001	1.32	1.15–1.52	<.001	1.32	1.15–1.52	<.001
Body Mass Index			<.001						
< 18.5	Ref								
18.5–25	1.03	0.72–1.46							
25–30	1.24	0.87–1.76							
30–40	1.64	1.15–2.33							
≥ 40	1.97	1.31–2.97							
Smoking			.184			<.001			.003
Yes	Ref			Ref			Ref		
No, never	0.99	0.90–1.09		0.78	0.69–0.87		0.82	0.72–0.92	
No, but ever	0.90	0.80–1.02		0.91	0.79–1.04		0.93	0.81–1.07	
Alcohol usage			<.001			.017			.012
Never	Ref			Ref			Ref		
Not in previous 12 months	0.88	0.77–1.02		0.96	0.82–1.12		0.95	0.81–1.12	
Monthly or less	0.68	0.61–0.77		0.83	0.72–0.95		0.82	0.71–0.94	
2–4 times per month	0.73	0.64–0.83		0.93	0.79–1.09		0.92	0.78–1.08	
2–3 times per week	0.64	0.55–0.75		0.92	0.76–1.10		0.89	0.74–1.08	
≥ 4 times per week	0.48	0.39–0.58		0.70	0.55–0.88		0.68	0.54–0.86	
Difficulty with Dutch language			<.001						
No	Ref								
Yes	1.32	1.21–1.43							
Not applicable	0.80	0.71–0.91							
Perceived health			<.001			<.001			.002
Excellent	Ref			Ref			Ref		
Very good	1.09	0.86–1.37		1.05	0.83–1.34		1.03	0.80–1.31	
Good	1.55	1.27–1.90		1.22	0.99–1.51		1.20	0.97–1.49	
Mediocre	2.47	2.01–3.04		1.37	1.09–1.72		1.32	1.05–1.67	
Bad	3.85	3.02–4.91		1.69	1.28–2.23		1.59	1.20–2.11	
Antibiotic-related behavior									
Higher antibiotic knowledge			<.001						
No	Ref								
Yes	0.77	0.71–0.84							
Ever asked GP for antibiotics			<.001						<.001
No, never	Ref						Ref		
Yes, regularly	4.72	3.59–6.21					3.07	2.28–4.14	
Yes, occasionally	2.42	2.20–2.66					2.11	1.91–2.34	
Did not finish treatment			<.001						<.001
Always finished or no antibiotics	Ref						Ref		
Yes, regularly	2.70	2.02–3.62					1.80	1.29–2.50	
Yes, occasionally	1.62	1.45–1.83					1.32	1.16–1.50	

*Abbreviations*: *OR* Odds Ratio, *aOR* adjusted Odds Ratio, *CI* Confidence Interval, *CVA* Cerebro Vascular Accident, *MI* Myocardial infarction, *GP* General Practitioner

Table 5 shows the results from the analysis on the association between ethnicity and total number of antibiotic prescriptions received in the year prior to the HELIUS study visit. Differences across ethnic groups were observed overall for the number of antibiotic prescriptions in both univariable and multivariable analysis (both *p* = 0.004). First-generation African Surinamese and Turkish migrants had a significantly lower number of antibiotic prescriptions compared to individuals of Dutch origin. Only second-generation Ghanaian participants has more prescriptions compared to Dutch participants. Furthermore, female sex, diabetes mellitus, MI, malignant disorder, respiratory disease, eczema and worse perceived health were significantly associated with a higher number of antibiotic prescriptions.

**Table 5:** Variables associated with number of antibiotic prescriptions in participants linked to ADH (*N* = 15,007) (zero-inflated negative binomial regression analysis)

	Univariable^a^			Multivariable excluding variables on antibiotic-related behavior			Multivariable including variables on antibiotic-related behavior		
	IRR	(95% CI)	*P*-values	IRR	(95% CI)	*P*-values	IRR	(95% CI)	*P*-values
Sociodemographics									
Ethnicity			.004			.004			.001
Dutch	Ref			Ref			Ref		
South-Asian Surinamese									
1st generation	1.06	0.85–1.31		0.86	0.68–1.10		1.02	0.82–1.28	
2nd generation	0.82	0.55–1.21		0.94	0.61–1.44		0.94	0.64–1.36	
African Surinamese									
1st generation	0.79	0.64–0.99		0.73	0.58–0.93		0.81	0.65–1.00	
2nd generation	0.83	0.57–1.22		0.90	0.59–1.36		0.93	0.65–1.33	
Ghanaian									
1st generation	0.75	0.60–0.94		0.77	0.60–1.00		0.81	0.65–1.02	
2nd generation	1.66	0.89–3.11		2.09	1.06–4.12		2.70	1.47–4.94	
Turkish									
1st generation	0.85	0.69–1.04		0.74	0.59–0.92		0.79	0.64–0.97	
2nd generation	1.14	0.87–1.51		1.14	0.84–1.53		1.10	0.84–1.44	
Moroccan									
1st generation	0.99	0.81–1.21		0.89	0.70–1.11		0.89	0.72–1.10	
2nd generation	0.84	0.63–1.13		0.92	0.67–1.27		1.02	0.76–1.37	
Female sex	1.36	1.20–1.54	<.001	1.35	1.18–1.54	<.001	1.29	1.15–1.46	<.001
Age			.023						
< 25 years	Ref								
25–34 years	1.09	0.91–1.30							
35–44 years	1.16	0.98–1.38							
45–54 years	1.09	0.92–1.29							
55–64 years	1.14	0.95–1.36							
≥ 65 years	1.44	1.16–1.80							
Educational level			.096						
Unknown	Ref								
No school/elementary school	1.74	0.86–3.52							
Lower vocational/lower secondary school	1.74	0.86–3.52							
Intermediate vocational/ intermediate secondary school	1.57	0.77–3.17							
Higher vocational/university	1.45	0.71–2.96							
Marital status			.212						
Married/registered partnership	Ref								
Cohabiting	0.85	0.72–1.00							
Unmarried/never married	0.99	0.89–1.10							
Divorced/separated	1.01	0.90–1.13							
Widow/widower	1.14	0.90–1.45							
Health status									
Self-reported medical conditions (previous 12 months)									
Diabetes mellitus	1.34	1.17–1.54	<.001	1.21	1.04–1.41	.015	1.22	1.06–1.41	.005
CVA/one-sided loss of bodily function ≤1 day	1.10	0.95–1.29	.208						
MI incl. ≥half hour chest pain or dotter/bypass operation	1.35	1.19–1.54	<.001	1.22	1.06–1.41	.005			
Severe heart condition	1.26	1.04–1.52	.019						
Malignant disorder	1.91	1.47–2.48	<.001	1.60	1.21–2.12	.001	1.60	1.28–2.00	
Severe or chronic fatigue	1.29	1.15–1.44	<.001						
High blood pressure	1.14	1.04–1.25	.006						
Artery stenosis	1.30	1.08–1.57	.005						
Respiratory diseases	1.50	1.32–1.71	<.001	1.34	1.16–1.54	<.001	1.29	1.13–1.47	<.001
Serious/persistent intestinal disorders	1.24	1.07–1.44	.004						
Psoriasis	1.08	0.90–1.31	.406						
(Chronic) eczema	1.21	1.07–1.37	.002	1.14	1.00–1.29	.042			
Incontinence	1.31	1.13–1.50	<.001						
Body Mass Index			.736						
< 18.5	Ref								
18.5–25	0.98	0.70–1.37							
25–30	0.98	0.70–1.37							
30–40	1.04	0.74–1.46							
≥ 40	1.06	0.71–1.57							
Smoking			.099						
Yes	Ref								
No, never	1.13	1.00–1.29							
No, but ever	1.02	0.87–1.21							
Alcohol usage			.075						
Never	Ref								
Not in previous 12 months	0.93	0.77–1.13							
Monthly or less	0.94	0.80–1.11							
2–4 times per month	1.01	0.85–1.20							
2–3 times per week	0.73	0.58–0.91							
≥ 4 times per week	0.81	0.61–1.08							
Difficulty with Dutch language			.320						
No	Ref								
Yes	0.94	0.86–1.04							
Not applicable	1.05	0.88–1.25							
Perceived health			<.001			.001			<.001
Excellent	Ref			Ref			Ref		
Very good	0.98	0.68–1.42		1.03	0.71–1.49		0.99	0.70–1.39	
Good	1.29	0.94–1.77		1.19	0.85–1.65		1.17	0.86–1.59	
Mediocre	1.73	1.25–2.39		1.40	1.00–1.97		1.44	1.05–1.97	
Bad	2.30	1.62–3.26		1.71	1.17–2.49		1.72	1.22–2.43	
Antibiotic-related behavior									
Higher antibiotic knowledge			.054						
No	Ref								
Yes	0.92	0.85–1.00							
Ever asked GP for antibiotics			<.001						<.001
No, never	Ref						Ref		
Yes, regularly	2.96	2.34–3.73					1.87	1.45–2.41	
Yes, occasionally	1.75	1.59–1.92					1.15	1.06–1.30	
Did not finish treatment			<.001						
Always finished or no antibiotics	Ref								
Yes, regularly	1.50	1.12–2.00							
Yes, occasionally	1.32	1.18–1.48							

*Abbreviations*: *IRR* Incidence Risk Ratio, *CI* Confidence Interval, *CVA* Cerebro Vascular Accident, *MI* Myocardial infarction, *GP* General Practitioner

^a^Accounts for zero-inflated distribution

Having a higher level of antibiotic knowledge was not significantly associated with the number of prescriptions when included in multivariable analysis (*p* = 0.446). No significant interactions between ethnicity and sex or education were observed. Finally, adjusting the association between ethnicity and antibiotic use for antibiotic-related behaviors did not change these associations.

## Discussion

Our study shows that knowledge on the need to use antibiotics for treatment is lower among all ethnic minority groups compared to Dutch, with second generation ethnic minorities showing higher levels of knowledge compared to first generation migrants. We also observed ethnic differences in the use of antibiotics, with a higher proportion having received at least one prescription, but a lower mean number of antibiotic prescriptions among some ethnic minority groups compared to Dutch. The only ethnic group with a significantly higher number of antibiotic prescriptions was second generation Ghanaian participants. Furthermore, we showed that a lower level of antibiotic knowledge was not associated with receiving antibiotics or average number of antibiotic prescriptions, and that ethnic differences in antibiotic use therefore cannot be explained by level of knowledge on antibiotics.

A previous study in Dutch primary care centres demonstrated higher use of antibiotics among first-generation migrants from Turkey, Morocco, Surinam or the Antilles compared to Dutch, after adjustment for age, sex, education, presence of chronic diseases, and smoking [3]. We found that the odds of having ≥1 antibiotic prescription was higher in some ethnic groups in unadjusted analysis, but after adjusting for several variables including medical conditions, the odds were significantly higher among Ghanaian and first-generation Moroccan participants only. In contrast, in our analyses on the number of antibiotic prescriptions as an outcome, only second-generation Ghanaian migrants were at higher risk of receiving a higher number of prescriptions compared to Dutch participants. For all other ethnic groups, no evidence of a higher risk for more frequent prescriptions was found, while even a lower number was present for first-generation African Suriname and Turkish participants. To the best of our knowledge, no other studies have evaluated the variation in level of antibiotic knowledge and antibiotic use between ethnic groups and thus our findings need to be confirmed. Notably, our findings on antibiotic prescriptions and ethnicity are in line with a large retrospective cohort study performed in pediatric emergency departments in the United States [15]. This study also looked at the association between ethnicity and antibiotic prescribing, showing that other ethnic groups received less antibiotics for viral infections than non-Hispanic white children.

Lower odds for higher level of antibiotic use knowledge were also seen among individuals who regularly or occasionally requested antibiotics from their GP or who regularly or occasionally did not finish treatment. These findings suggest that improving antibiotic knowledge might decrease the number of requests for antibiotics in primary care and improve appropriate use.

Our study has several strengths. First, the HELIUS study consists of a large number of participants from major ethnic groups living in the same city, with representation from all socioeconomic levels. Second, all outcomes and determinants were measured using the same methodology across all ethnic groups and HELIUS used translated questionnaires and had ethnically-matched interviewers and research assistants to provide assistance during data collection. These procedures enhance the comparability between ethnic groups. Another major strength of the current study is that HELIUS data could be linked to data from a health insurance register covering the majority (77.7%) of the study population.

Our study has also limitations. First, although HELIUS participants were recruited via an ethnicity-stratified random selection of the municipal registry of Amsterdam, the response rate for HELIUS study was 28% and there may be selection bias [8]. However, analysis from a previous HELIUS study have shown that participants are not exceedingly different from non-respondents regarding sociodemographic variables [8]. Second, we did not take into account the use of antibiotics purchased over the counter in the home country of participants [6, 16–18], and we might therefore have underestimated antibiotic use in non-Dutch ethnic groups. As a recent HELIUS study found that Dutch people of Turkish or Moroccan origin were more likely to use healthcare in the Netherlands as well as their country of origin [19], underestimation of antibiotic use in non-Dutch ethnic groups seems unlikely. Third, since several characteristics, such as education level and medical conditions, of HELIUS participants insured at Achmea differed from those insured elsewhere, selection bias could have been introduced in analysis on antibiotic use. This difference could be due to the fact that the City of Amsterdam provided health insurance discounts with Achmea for low-income individuals. These differences were corrected for during multivariable analyses to the most possible extent. Fourth, the variable ‘ever asked GP for antibiotics’ does not discriminate between appropriate or inappropriate requests for antibiotics and misclassification might have occurred. However, this variable gives some information on participants’ attitudes towards antibiotic use. Furthermore, due to privacy restrictions, we were unable to include indication for antibiotic therapy and duration of antibiotic use as additional indices for antibiotic use (apart from the number of antibiotics prescribed). Moreover, since this was a cross-sectional study, we were unable to model antibiotic knowledge with future antibiotic prescriptions. Further research should examine the association of antibiotic knowledge with future antibiotic prescriptions. Finally, we are unable to determine if individuals were more demanding towards their GP or if their GPs were more lenient in prescribing antibiotics during illness [4, 5]. Neither completing antibiotic therapy, assessed by pill count, nor duration of antibiotic use could be taken into account as these data were not available.

## Conclusions

To our knowledge, this study is the first to examine ethnic disparities in level of antibiotic knowledge and use in a large population-based sample among adults with different ethnic backgrounds. Health policy makers and healthcare professionals are increasingly developing interventions to improve the quality of antibiotic use, which is needed to help contain antimicrobial resistance. Targeted campaigns can be considered, for instance, during the annual European Antibiotic Awareness Day, since this event addresses improvement in the quality of antibiotic use to the general public [20]. Still, this study shows that a lower level of antibiotic knowledge is not necessarily linked to higher antibiotic usage, indicating that interventions aimed at improving knowledge alone might be insufficient to reduce antibiotic use. Nevertheless, the underlying reasons for these findings need further evaluation.

## Supplementary information

**Additional file 1.** Supplementary Methods.

**Additional file 2:** **Table S1.** Characteristics of participants not linked versus linked to the Achmea Health Database.

## Data Availability

The datasets used in the current study are available from the corresponding author on request, contingent on approval from the HELIUS scientific committee.

## Abbreviations

AHD: Achmea Health Database
aIRR: adjusted Incidence Risk Ratio
aOR: adjusted Odds Ratio
BMI: Body Mass Index
CVA: Cerebrovascular Accident
GP: General Practitioner
HELIUS: Healthy life in an Urban Setting
IQR: Interquartile Range
IRR: Incidence Risk Ratio
MI: Myocardial Infarction
OR: Odds Ratio
WHO: World Health Organization
