# Supplementary material for: Knowledge and use of antibiotics in six ethnic groups: the HELIUS study
Source: Antimicrob Resist Infect Control. 2019 Dec 6;8:200. doi: 10.1186/s13756-019-0636-x (PMC6898914; doi:10.1186/s13756-019-0636-x)
Supplement: Supplementary file 1 — Additional file 1. Supplementary Methods. [file 13756_2019_636_MOESM1_ESM.docx]

**SUPPLEMENTARY METHODS**

**Determining threshold to define knowledge level of antibiotic use**

In order to define antibiotic knowledge, we used an approach based on item response theory (IRT) (1). In brief, IRT allows a more precise definition of the relationship between a given measurement process (defined by questions related to correct antibiotic use for specific illnesses) and a latent trait (defined as ‘higher’ or ‘lower’ antibiotic knowledge). The latent space is assumed to be unidimensional (i.e. only one latent trait is being measured) and conditional independence is assumed between item responses.

The probability of an individual to correctly answer a question on antibiotic use is in function of the latent trait, *θ*, and the item parameters. Each item has two parameters: discrimination (correlation between correct item response and *θ*, with larger values representing capacity to differentiate between low and high levels of the latent trait) and difficulty (describing the location of an item with respect *θ*, with larger values indicating that the expected probability of a correct answer corresponds to higher levels of the latent trait).

We first modeled discrimination and difficulty parameters (defined as *a* and *b*, respectively) for each binary item using a one-parameter logistic model, whereby *a* is shared across all items and *b* is allowed to vary between items. This model was compared to a two-parameter logistic model, whereby both *a* and *b* are allowed to vary between items, using a likelihood-ratio test. After selecting the appropriate IRT logistic model, the probability of correctly answering each individual item was plotted in function of *θ* [defined as an item characteristic curve (ICC)].

We created an overall knowledge score on antibiotic use by summing the total number of correct responses, resulting in a score ranging from 0-5. In order to plot the expected score across levels of *θ*, a total characteristic curve (TCC) was constructed from summing the ICCs across all items. We defined higher” and “lower” antibiotic knowledge as a score corresponding to a *θ* = 0. The analysis was carried out using the “irt” commands in Stata (v15.0, College Station, TX).

**Reference:**

1. Linden WJ van der, editor. Handbook of item response theory. New York: CRC Press; 2015. (Statistics in the Social and Behavioral Sciences).

**SUPPLEMENTARY RESULTS**

**Threshold for higher knowledge of antibiotic use**

The one-parameter IRT logistic model resulted in discrimination parameter *a* = 0.54 (95%CI= 0.51, 0.57) and difficulty parameter *b* = -3.16 (95%CI=-3.32, -2.99) for the item on influenza, -2.84 (95%CI=-2.99, -2.69) for fever, -2.34 (95%CI=-2.47, -2.21) for pneumonia, -2.08 (95%CI=-2.20, -1.96) for sore throat, and -0.50 (95%CI=-0.56, -0.44) for bronchitis.

The two-parameter IRT logistic model resulted in the following discrimination and difficulty parameters, respectively: *a* = -0.75 (95%CI=-0.81, -0.69) and *b* = 1.77 (95%CI=1.64, 1.91) for the item on pneumonia, *a* = 0.62 (95%CI=0.57, 0.67) and *b* = -0.44 (95%CI=-0.49, -0.38) for bronchitis, *a* = 1.53 (95%CI=1.44, 1.62) and *b* = -0.96 (95%CI=-1.00, -0.92) for sore throat *a* = 2.05 (95%CI=1.91, 2.18) and *b* = -1.25 (95%CI=-1.30, -1.21) for influenza, *a* = 2.25 (95%CI=2.09, 2.41) and *b* = -1.09 (95%CI=-1.13, -1.05) for fever.

The two-parameter IRT logistic model was tested against the one-parameter model, with the former demonstrating better fit according to the likelihood ratio test (*p*<0.001). The resulting ICC is shown in Figure S1A. Given the ICC, the item on antibiotic use during pneumonia would not be regarded as useful in determining the latent trait of this study population. Nevertheless, this question remained in the composite score in order to be consistent with previous studies. The resulting TCC is shown in Figure S1B, a score of 4.02 (rounded to 4) or higher corresponds to a *θ* ≥ 0 and hence was determined as the threshold in defining “higher” knowledge on antibiotic use.

**SUPPLEMENTARY FIGURES**

**Figure S1. Item characteristic curve (A) and total characteristic curve (B) on knowledge of antibiotic use**

| **A** |  |
| --- | --- |
| **B** |  |

In the item characteristic curve (**A**), the probability of answering an item correctly, Pr(item=1), is plotted against levels of the latent trait, theta. Item parameters can be obtained from this graph, with discrimination being the instantaneous slope and difficulty the corresponding theta when Pr(item=1) is 0.5. In the total characteristic curve (**B**), the expected score is plotted against levels of the latent trait, theta.
