# Supplementary material for: Knowledge and use of antibiotics in six ethnic groups: the HELIUS study
Source: Antimicrob Resist Infect Control. 2019 Dec 6;8:200. doi: 10.1186/s13756-019-0636-x (PMC6898914; doi:10.1186/s13756-019-0636-x)
Supplement: Supplementary file 2 — Additional file 2: Table S1. Characteristics of participants not linked versus linked to the Achmea Health Database. [file 13756_2019_636_MOESM2_ESM.docx]

Supplementary table 1. Characteristics of participants not linked versus linked to the Achmea Health Database

|  | | **Non-linked participants**  **(N=6,956)** | | **Linked  participants**  **(N=15,007)** | | **P-value** |
| --- | --- | --- | --- | --- | --- | --- |
|  | | n | % | n | % |  |
| **Sociodemographics** | |  |  |  |  |  |
| **Ethnicity** | |  |  |  |  | <.001 |
| Dutch | | 2,493 | 35% | 2,071 | 14% |  |
| South-Asian Surinamese | | 946 | 13% | 2,097 | 14% |  |
| African Surinamese | | 1,385 | 19% | 2,766 | 18% |  |
| Ghanaian | | 466 | 7% | 1,873 | 12% |  |
| Turkish | | 736 | 10% | 2,878 | 19% |  |
| Moroccan | | 930 | 13% | 2,976 | 20% |  |
| Other/unknown | | 202 | 2.8% | 346 | 2.3% |  |
| **Female sex** | | 4,041 | 56% | 8,769 | 58% | .005 |
| **Median age in years (IQR)** | | 44 | (32-54) | 46 | (35-55) | <.001 |
| **Migration generation** | |  |  |  |  | <.001 |
| 1^st^ generation | | 3,436 | 48% | 10,283 | 69% |  |
| 2^nd^ generation | | 1,229 | 17% | 2,653 | 18% |  |
| Not applicable | | 2,493 | 35% | 2,071 | 14% |  |
| **Educational level** | |  |  |  |  | <.001 |
| Unknown | | 50 | 0.7% | 157 | 1.1% |  |
| No school/elementary school | | 621 | 9% | 3,255 | 22% |  |
| Lower vocational/lower secondary school | | 1,370 | 19% | 4,433 | 30% |  |
| Intermediate vocational/ intermediate secondary school | | 1,998 | 28% | 4,423 | 29% |  |
| Higher vocational/university | | 3,119 | 44% | 2,739 | 18% |  |
| **Marital status** | |  |  |  |  | <.001 |
| Married/registered partnership | | 2,689 | 38% | 5,910 | 40% |  |
| Cohabiting | | 1,031 | 14% | 1,377 | 9% |  |
| Unmarried/never married | | 2,559 | 36% | 4,929 | 33% |  |
| Divorced/ separated | | 751 | 11% | 2,357 | 16% |  |
| Widow/widower | | 106 | 1.5% | 328 | 2.2% |  |
| **Health status** | |  |  |  |  |  |
| **Self-reported medical conditions (previous 12 months)** | |  |  |  |  |  |
| Diabetes mellitus | | 396 | 6% | 1,606 | 11% | <.001 |
| CVA/one-sided loss of bodily function ≤1 day | | 304 | 4.3% | 837 | 6% | <.001 |
| MI incl. ≥half hour chest pain or dotter/bypass operation | | 610 | 9% | 1,902 | 13% | <.001 |
| Severe heart condition | | 126 | 1.8% | 467 | 3.1% | <.001 |
| Malignant disorder | | 122 | 1.7% | 301 | 2.0% | .120 |
| Severe or chronic fatigue | | 1,596 | 22% | 4,395 | 30% | <.001 |
| High blood pressure | | 1,147 | 16% | 3,378 | 23% | <.001 |
| Artery stenosis | | 234 | 3.3% | 907 | 6% | <.001 |
| Respiratory diseases | | 602 | 8% | 1,708 | 11% | <.001 |
| Serious/persistent intestinal disorders | | 495 | 7% | 1,231 | 8% | .001 |
| Psoriasis | | 238 | 3.3% | 560 | 3.8% | .124 |
| (Chronic) eczema | | 698 | 10% | 1,532 | 10% | .265 |
| Incontinence | | 523 | 7% | 1,374 | 9% | <.001 |
| **Median Body Mass Index (kg/m^2^) (IQR)** | | 25.2 | (22.6-28.5) | 26.9 | (23.9-30.6) | <.001 |
| **Smoking** | |  |  |  |  | <.001 |
| Yes | | 1,734 | 24% | 3,568 | 24% |  |
| No, never | | 3,735 | 52% | 8,600 | 58% |  |
| No, but ever | | 1,664 | 23% | 2,757 | 18% |  |
| **Alcohol usage** | |  |  |  |  | <.001 |
| Never | | 2,090 | 29% | 6,927 | 46% |  |
| Not in previous 12 months | | 439 | 6% | 1,361 | 9% |  |
| Monthly or less | | 1,123 | 16% | 2,457 | 16% |  |
| 2-4 times per month | | 1,238 | 17% | 1,817 | 12% |  |
| 2-3 times per week | | 1,221 | 17% | 1,322 | 9% |  |
| ≥4 times per week | | 1,024 | 14% | 1,019 | 7% |  |
| **Difficulty with Dutch language** | | 1,314 | 18% | 5,857 | 39% | <.001 |
| **Perceived health** | |  |  |  |  | <.001 |
| Excellent | | 629 | 9% | 922 | 6% |  |
| Very good | | 1,539 | 22% | 2,020 | 14% |  |
| Good | | 3,755 | 53% | 7,662 | 51% |  |
| Fair | | 1,064 | 15% | 3,605 | 24% |  |
| Poor | | 154 | 2.2% | 754 | 5% |  |
| **Antibiotics** | |  |  |  |  |  |
| **Knowledge concerning antibiotics** | |  |  |  |  |  |
| Antibiotics effective for influenza | | 909 | 13% | 2,690 | 19% | <.001 |
| Antibiotics effective for pneumonia | | 5,810 | 82% | 10,816 | 74% | <.001 |
| Antibiotics effective for fever | | 1,224 | 18% | 2,879 | 20% | <.001 |
| Antibiotics effective for sore throat | | 1,574 | 22% | 3,993 | 27% | <.001 |
| Antibiotics effective for bronchitis | | 3,263 | 47% | 6,123 | 42% | <.001 |
| **Higher antibiotic knowledge*** | | 5,050 | 73% | 9,593 | 67% | <.001 |
| **Did not finish treatment** | |  |  |  |  | <.001 |
| Yes, regularly | | 48 | 0.7% | 193 | 1.3% |  |
| Yes, occasionally | | 656 | 9% | 1,618 | 11% |  |
| **Saved antibiotics for later** | |  |  |  |  | <.001 |
| Yes, regularly | | 6 | 0.1% | 33 | 0.2% |  |
| Yes, occasionally | | 97 | 1.4% | 224 | 1.5% |  |
| Not applicable | | 6,396 | 90% | 12,962 | 88% |  |
| **Ever asked GP for antibiotics** | |  |  |  |  | .001 |
| Yes, regularly | | 62 | 0.9% | 212 | 1.4% |  |
| Yes, occasionally | | 1,198 | 17% | 2,568 | 17% |  |
| * | Based on a summed score with cutoff determined by an Item Response Theory model (≥4 out of 5 antibiotic knowledge questions correctly answered was considered higher level of knowledge) | | | | | |
|  | Missing observations on the following variables: marital status 128; diabetes 78; stroke 97; myocardial infarction 193; heart condition 83; malignant disorders 137; migraine 113; fatigue 145; high blood pressure 101; artery stenosis 140; respiratory diseases 115; bowel diseases 115; psoriasis 98; eczema 117; incontinence 126; BMI 23; smoking 107; alcohol 127; perceived health 61; AB effective for influenza 614; AB effective for pneumonia 477; AB effective for fever 685; AB effective for sore throat 625; AB effective for bronchitis 663; higher antibiotic knowledge 958; asked GP for AB 414; did not finish treatment 292; saved AB 325 | | | | | |
|  | Abbreviations: IQR – Inter Quartile Range; CVA – Cerebro Vascular Accident; MI – Myocardial infarction; GP – General Practitioner | | | | | |
